# Is obesity a contraindication for simultaneous bilateral total knee arthroplasty? A prospective case–control study

**DOI:** 10.1051/sicotj/2020040

**Published:** 2020-10-30

**Authors:** Sanjay Agarwala, Yash Santosh Wagh, Mayank Vijayvargiya

**Affiliations:** 1 Chief of Orthopaedics and Director Professional Services, P.D Hinduja Hospital and Medical Research Centre Mumbai India; 2 Clinical Assistant, P.D Hinduja Hospital and Medical Research Centre Mumbai India; 3 Junior Consultant, Department of Orthopedics, P.D Hinduja Hospital and Medical Research Centre Mumbai India

**Keywords:** Total Knee Arthroplasty, Obesity, Oxford Knee Score, Complication rate

## Abstract

*Aim*: Total Knee Arthroplasty (TKA) for decades has been an effective treatment modality for chronic arthritis of the knee. However, there is scarcity of literature comparing the functional outcomes of simultaneous bilateral TKA in obese patients with non-obese Indian population. We conducted this study to evaluate the functional outcomes and complication rates of simultaneous bilateral TKA in obese patients matched control with non-obese patients. *Materials and methods*: We divided the patients into two study groups based on their body mass index (BMI). Patients with a BMI of less than 30 were classified as non-obese and those with a BMI of more than 30 were classified as obese. All the patients underwent simultaneous bilateral TKA by a single surgeon using the same implant and technique. Patients were followed up regularly and functional outcomes in terms of Oxford knee score were noted at 6 weeks, 3 months, and 1 year. Post-operative complications and time to recovery was also compared. *Results*: Mean follow-up in obese group was 18 months (12–25 months) and in non-obese group was 17 months (12–24 months). Both the groups were matched with control in terms of pre-operative parameters. Post-operative hemoglobin drop, ICU requirement, length of hospital stay, mean walking time, and mean time to climbing stairs were similar in both the groups. Oxford knee score was significantly better in non-obese group at 6 weeks, but was similar in both the groups at 3 months, 6 months, 1 year, and last follow-up. There was no statistically significant difference seen in the complication rate in both the groups. There was no implant loosening or radiolucency seen. *Conclusion*: We conclude in our study that simultaneous bilateral TKA gives comparable mid-term results in obese patients in comparison to the non-obese patients.

## Introduction

Over the past two decades, total knee arthroplasty (TKA) has proved to be an effective solution for chronic knee pain arising due to arthritis [1]. It has been offered to obese patients who were considered a relative contraindication a few years ago [2]. In the modern era, TKA as a surgery is being performed very commonly in the obese population. The WHO, defines obesity as BMI of above 30 kg/m^2^ [3]. Numerous studies have shown a correlation between elevated BMI resulting in articular cartilage loading forces, which may eventually cause tissue damage and early joint degeneration in the obese population [4].

Total knee arthroplasty in obese patients has its challenges starting from associated co-morbidities, to difficulties faced during surgery, such as positioning, exposure, increased chances of intraoperative bleeding, avulsion of the medial collateral ligament, and delayed recovery [5]. Studies conducted in the past reveal that obesity has an adverse effect on the final clinical outcome of obese patients undergoing TKA, such as delayed wound healing, higher medical complications, higher infection rates, poor post-operative functional out comes, and, ultimately, delayed recovery [5–8].

There have been many studies published on the outcome of TKA in obese patients, but still, it is inconclusive whether TKA has similar results in obese patients in terms of clinical outcome in comparison to non-obese patients [9–15]. A vast majority of the studies that are published in western literature are retrospective, and there is a scarcity of literature comparing the final outcomes of TKA in obese and non-obese patients, managed with the same protocol as in the Indian population [16–18]. Furthermore, none of the studies specifically has evaluated the outcomes of simultaneous bilateral TKA in obese patients as compared to non-obese patients. Therefore, it is still not clear whether simultaneous bilateral TKA should be offered to obese patients having bilateral Osteoarthritis (OA) knee; or simultaneous bilateral TKA in obese patients will have more complications or poorer outcomes as compared to non-obese patients.

Therefore, we have conducted this prospective cohort study to compare the results of TKA in obese and non-obese patients. The groups of subjects were matched in the Indian scenario to evaluate whether simultaneous bilateral TKA has any effect on the functional outcome, recovery, and complication rate in obese patients in comparison to non-obese patients. This is the only study that has compared the functional outcomes of obese patients who underwent bilateral simultaneous TKA and matched control with non-obese bilateral simultaneous TKA patients.

## Materials and methods

This study is a prospective research model where we analyzed 52 patients divided equally into two groups comprising of 26 obese and 26 non-obese patients who were operated for bilateral TKA between January 2018 and June 2019. Detailed history regarding symptoms, co-morbidities, and demographics was noted and BMI was calculated by dividing each patient’s weight in kilograms (kg) by square of the calculated height in meters (m) Kg/m^2^ (Table 1). The patient groups, in our study, were divided into two with subjects having Body Mass Index (BMI) less than 30 classified as non-obese and subjects with BMI 30–40 classified as obese patients. We included patients between the age of 40 and 75 years, who were willing to undergo bilateral simultaneous TKA for arthritis of the knee joint. All patients undergoing revision surgery, with a history of post-traumatic arthritis, history of knee joint infection, and morbidly obese patients with a BMI of more than 40 were excluded from our study. The preoperative comorbidities were similar and comparable in both the groups, and all patients fit to undergo surgery were only considered in our study.

**Table 1:** Pre-operative demographics’ comparison of the two groups.

	Obese	Non-obese	*P* value < 0.05 significant
Parameters	Mean ± SD	Mean ± SD	
Age	64.44 ± 7.89	65.31 ± 8.87	0.71
Gender			
Male	14.81%	26.92%	0.27
Female	85.19%	73.08%	
BMI	34.43 ± 2.42	26.08 ± 3.65	1.64
Arthritis			
OA	92.59%	88.46%	0.60
RA	7.41%	11.54%	
^*^ASA			
ASA1	44.44%	34.62%	0.76
ASA2	51.85%	61.54%	
ASA3	3.71%	3.84%	
ASA4	0%	0%	
Diabetes			
Yes	18.52%	34.62%	0.18
No	81.48%	65.38%	
Cardiac comorbidity			
Yes	7.41%	11.54%	0.18
No	92.59%	88.46%	
Hyertension			
Yes	33.33%	23.08%	0.40
No	66.67%	76.92%	
Pre Op Hemoglobin	12.76 ± 0.92	13.12 ± 1.39	0.27

* ASA – American Society of Anesthesiologists.

All the cases were operated by a single surgeon by a standard parapatellar approach all implants by the same manufacturer, that is, Smith and Nephew posterior stabilized high flexion genesis 2 system. Patelloplasty was performed in each of the patients in our study group [19]. We have used our technique of Concealed cosmetic closure for all patients [20].

All patients were made to walk full weight-bearing from the first post-operative day and the knee range of motion was started. All patients were given deep venous thrombosis (DVT) by means of mechanical calf pumps, above knee stockings early mobilization and anticoagulant therapy i.e., low molecular weight heparin (LMWH). The average time of discharge was day 3–4 post operatively.

Patients were followed up at two weeks (to assess any early wound related complications), six weeks, three months, one year, and annually thereafter. The oxford knee scores of the patients were noted at each follow-up and compared to their pre-operative scores; any complications such as DVT, superficial or deep infections, pain and any other complication were appropriately treated and recorded. Radiological evaluation was done at each follow-up to look for any signs of loosening, implant failure, and signs of infection such as osteolysis

### Statistical analysis

The sample size of 52 patients was determined by convenience sampling using the Sample Size Calculator − The Survey System. Data recording was done using MS Excel. Descriptive statistics for quantitative variables (age, height, weight, BMI) were represented as mean ± SD. Qualitative variables were represented as frequency and percentages. Statistical comparisons of categorical variables were done using Chi-Square analysis. For non-normally distributed quantitative data, the Mann-Whitney U test (two groups with Oxford Knee Score) was used. All tests were two-tailed, and the results were considered significant at *P* ≤ 0.05.

## Results

The proportion of age, sex, deformity of limbs, associated co-morbidities, type of anesthesia administered, intra-operative blood loss, intra-operative and post-operative complications, duration of surgery, days of admission, and rehabilitation were not statistically significant (Tables 1 and 2). The average age group undergoing total knee arthroplasty in our study was 64.8 years, with the obese patients being lower at an average age of 64.44 and the non-obese group having an age of presentation at 65.33, which was not significant. The mean follow-up in obese group was 18 months (12–25 months) and in non-obese group was 17 months (12–24 months). We compared the Oxford Knee Scores in each of our patients at 6 weeks, 3 months, and 1 year (Table 3). It was found that the OKS in both groups was statistically significant at 6 weeks with non-obese patients having a better functional outcome in both the knees than the obese group. The *P*-values, however, for both the knees at 3 months and 1 year were not significant suggesting good outcomes in both the groups.

**Table 2:** Post-operative parameters’ comparison of the two groups.

Parameters	Obese	Non-obese	*P* value
Post operative hemoglobin	11.13 ± 1.10	11.29 ± 1.74	0.68
Hemoglobin drop	1.64 ± 0.53	1.83 ± 0.69	0.59
Tourniquet time (min) total	101.44 ± 6.31	100.81 ± 7.41	0.90
Post operative ICU requirement			
Yes	3.70%	11.54%	0.28
No	96.30%	88.46%	
Length of hospital stay (days)	4.07 ± 0.62	4.15 ± 0.78	0.53
Walking time (days)	0.33 ± 0.48	0.19 ± 0.40	0.54
Climbing stairs (days)	1.48 ± 1.01	1.12 ± 1.03	0.53

**Table 3:** Comparison of the Oxford Knee Score between the two groups.

	Right knee			Left knee		
Oxford knee score	obese	Non-obese	*P* value	Obese	Non-obese	*P* value
Pre-operative	12.96 ± 2.59	14.35 ± 2.88	0.07	12.59 ± 2.48	13.96 ± 2.90	0.06
6 weeks	24.26 ± 5.01	36.69 ± 3.03	3.38	24.48 ± 3.91	37 ± 1.94	3.99
3 months	43.48 ± 1.62	43.31 ± 2.29	0.48	42.63 ± 2.08	43.04 ± 1.61	0.36
1 year	46.59 ± 1.62	46.69 ± 1.01	0.77	46.48 ± 1.48	46.31 ± 0.97	0.26
Last follow-up	47.82 ± 1.32	47.94 ± 1.58	0.85	47.36 ± 1.24	47.54 ± 1.14	0.34

### Complications

Deep vein thrombosis developed in one patient in the non-obese group was managed conservatively and was not significant. Four patients (two patients in each group) had acute urinary retention in the immediate post-operative period. Two patients (three knees – one patient had one side and the other bilaterally) in the obese group and one patient (two knees – bilaterally) in the non-obese group had superficial wound complications (*P* > 0.05). They were managed with thorough wound debridement. One patient each in obese with osteoarthritis (two knees-bilateral) and non-obese group (one knee-unilateral) had a patellofemoral problem post-operatively (*P* > 0.05). Both the patients underwent patella resurfacing. None of the patients had any signs of implant loosening/failure at the end of our study. We had one mortality in the obese group during our study at six months, the cause of death being congestive cardiac failure due to hypovolemic shock sustained after an accidental trauma, and was not related to the Bilateral TKR surgery.

## Discussion

We conducted a prospective study to analyze the long-term outcomes of bilateral total knee arthroplasty in obese and non-obese patients by a single operating surgeon. The ASA scores (American Society of Anesthesia) in both the groups had no significant difference (*P*-value of 0.76) suggesting that both the groups matched for the pre-operative surgical risk assessment. Both the groups were also matched in terms of other pre-operative parameters and demographics.

Spicer et al. [10] found no significant difference in the functional outcome following TKA between non-obese and obese patients when assessed using Oxford Knee Score at a mean follow-up of 75.9 months. A similar outcome was also observed in our study where post-operative functional scores using the Oxford Knee score were similar between the two groups at a follow-up of 3 months, 6 months, and at 1 year. Similarly, Griffin et al. [13] in their study of obese and non-obese patients found no difference in their post-operative outcomes at a 10-year follow-up; they stated that the knee scores and revision rates were similar in both the groups. Similar results have been reported by Amin et al. [14], patients where obese patients undergoing TKA had similar outcomes compared to non-obese patients. In our retrospective series of 402 TKA in 213 patients, we have observed that functional scores and complication rates in obese patients are comparable to the historical control of non-obese patients [21].

However, numerous studies have shown a negative impact of obesity on the functional outcome and complications rate following TKA. Foran et al. [9] reported poorer outcomes in obese population compared to the non-obese group. The authors concluded that for any degree of obesity, it has a negative effect on the outcome of total knee replacement. Yeung et al. [22] in their study of 50 obese TKA patients control-matched with non-obese TKA showed that although the obese group had lower post-operative knee scores, patient satisfaction, radiographic outcome, survivorship, and revision rates were similar in both the groups. Collins et al. [23] in their prospective study of 445 TKA demonstrated a slightly lower clinical outcome in highly obese (BMI ≥ 35 kg/m^2^) patients compared to non-obese (BMI < 30 kg/m^2^) and mild obese groups (BMI 30–35 kg/m^2^). Although there was no difference seen in the complication rates or survivorship between the groups, the authors concluded that given the substantial relief of symptoms after TKA and low complication and revision rates, they have found no reason to limit TKR in obese patients.

Literature is still inconclusive whether obese patients have more complications following TKA as compared to non-obese patients. Stern and Insall [12] in a fairly large study group of 257 patients have reported almost 30% of patients with symptomatic patellofemoral joints; however, 11% of these patients had a BMI of above 42. A study by Griffin et al. [13] also reported a high incidence of patellofemoral symptoms in the obese sub group. These studies have a higher incidence of patellofemoral complications due to the use of older implant designs. Using the technique of patellaplasty, the incidence of patellofemoral pain was significantly lowered and was observed in only two patients in our study.

Dowsey et al. [24] in their prospective study of 529 TKA concluded that the incidence of adverse effects is significantly higher in morbidly obese patients (35.1%) compared to obese (22.1%) and non-obese (14.2%) patients. The study also stated that with an increase in BMI, there was an increase in adverse effects by 8% in the obese population. Other studies have also reported significantly higher peri-operative and postoperative complications in obese patients undergoing TKR [25]. McElroy et al. [7] have clearly demonstrated an increased complication rate with almost 22% in the morbidly obese group, 15% in the obese, and only 9% in the non-obese group. Many studies report similar complication rates in obese patients [22, 23]. Amin et al. [14] in a study of 367 patients with a five-year follow-up showed no difference between the complication rates in the non-obese and obese groups. The complication rates in our study are comparable to their study.

## Conclusion

Bilateral single-stage total knee arthroplasty gives very good results in obese population providing them with an improved quality of life and excellent post-operative function. Our study showed that there were no significant intraoperative or postoperative complications in both obese and non-obese groups; hence, it is advisable to do this surgery even in obese patients for very encouraging results.

## Conflicts of interest

The authors received no financial support for the research, authorship, and/or publication of this article and there are no conflicts of interest.

## References

[1] Wylde V, Dieppe P, Hewlett S, Learmonth ID (2007) Total knee replacement: Is it really an effective procedure for all? Knee 14 (6), 417–423. 17596949 10.1016/j.knee.2007.06.001

[2] Pachore JA, Vaidya SV, Thakkar CJ, Bhalodia HKP, Wakankar HM (2013) ISHKS joint registry: A preliminary report. Indian J Orthop 47 (5), 505–509. 24133312 10.4103/0019-5413.118208 PMC3796925

[3] Obesity and overweight, World Health Organization Available at http://www.who.int/mediacentre/factsheets/fs311/en/index.html. May 2012.

[4] Coggon D, Reading I, Croft P, McLaren M, Barrett D, Cooper C (2001) Knee osteoarthritis and obesity. Int J Obes Relat Metab Disord 25, 622–627. 11360143 10.1038/sj.ijo.0801585

[5] Felson DT, Anderson JJ, Naimark A, Walker AM, Meenan RF (1988) Obesity and knee osteoarthritis. The Framingham Study. Ann Intern Med 109, 18–24. 3377350 10.7326/0003-4819-109-1-18

[6] Ayyar V, Burnett R, Coutts FJ, van der Linden ML, Mercer TH (2012) The influence of obesity on patient reported outcomes following total knee replacement. Arthritis. 185208 (6), 2012. 10.1155/2012/185208 PMC3483674 23119158

[7] McElroy MJ, Pivec R, Issa K, Harwin SF, Mont MA (2013) The effects of obesity and morbid obesity on outcomes in TKA. J Knee Surg 26 (2), 83–88. 23479424 10.1055/s-0033-1341407

[8] Rajgopal V, Bourne RB, Chesworth BM, et al. (2008) The impact of morbid obesity on patient outcomes after total knee arthroplasty. J Arthroplasty 23, 795–800. 18534516 10.1016/j.arth.2007.08.005

[9] Foran JR, Mont MA, Etienne G, Jones LC, Hungerford DS (2004) The outcome of total knee arthroplasty in obese patients. J Bone Joint Surg Am. 86–A(8), 1609–1615. 10.2106/00004623-200408000-00002 15292406

[10] Spicer DD, Pomeroy DL, Badenhausen WE, Schaper LA Jr, Curry JI, Suthers KE, Smith MW (2001) Body mass index as a predictor of outcome in total knee replacement. Int Orthop 25, 246–249. 11561501 10.1007/s002640100255 PMC3620835

[11] Mont MA, Mathur SK, Krackow KA, Loewy JW, Hungerford DS (1996) Cementless total knee arthroplasty in obese patients. A comparison with a matched control group. J Arthroplasty 11, 153–156. 8648308 10.1016/s0883-5403(05)80009-9

[12] Stern SH, Insall JN (1990) Total knee arthroplasty in obese patients. J Bone Joint Surg Am 72, 1400–1404. 2229120

[13] Griffin FM, Scuderi GR, Insall JN, Colizza W (1998) Total knee arthroplasty in patients who were obese with 10 years followup. Clin Orthop 356, 28–33. 10.1097/00003086-199811000-00006 9917664

[14] Amin AK, Patton JT, Cook RE, Brenkel IJ (2006) Does obesity influence the clinical outcome at five years following total knee replacement for osteoarthritis? J Bone Joint Surg [Br] 88-B, 335–340. 10.1302/0301-620X.88B3.16488 16498007

[15] Anandacoomarasamy A, Caterson I, Sambrook P, Fransen M, March L (2008) The impact of obesity on the musculoskeletal system. Int J Obes (Lond) 32, 211–222. 17848940 10.1038/sj.ijo.0803715

[16] Spector TD, Hart DJ, Doyle DV (1994) Incidence and progression of osteoarthritis in women with unilateral knee disease in the general population: the effect of obesity. Ann Rheum Dis 53, 565–568. 7979593 10.1136/ard.53.9.565 PMC1005406

[17] Szoeke C, Dennerstein L, Guthrie J, Clark M, Cicuttini F (2006) The relationship between prospectively assessed body weight and physical activity and prevalence of radiological knee osteoarthritis in postmenopausal women. J Rheumatol 33, 1835–1840. 16881096

[18] Anandacoomarasamy A, Fransen M, March L (2009) Obesity and the musculoskeletal system. Curr Opin Rheumatol 21, 71–77. 19093327 10.1097/bor.0b013e32831bc0d7

[19] Agarwala S, Shetty V, Karumuri LK, Vijayvargiya M (2018) Patellar resurfacing versus nonresurfacing with patellaplasty in total knee arthroplasty. Indian J Orthop 52 (4), 393–398. 30078898 10.4103/ortho.IJOrtho_512_16 PMC6055458

[20] Agarwala S, Vijayvargiya M (2019) Concealed cosmetic closure in total knee replacement surgery – A prospective audit assessing appearance and patient satisfaction. J Clin Orthop Trauma 10 (1), 111–116. 30705543 10.1016/j.jcot.2017.11.002 PMC6349645

[21] Agarwala S, Jadia C, Vijayvargiya M (2020) Is obesity a contra-indication for a successful total knee arthroplasty? J Clin Orthop Trauma 11(1), 136–139. 32002002 10.1016/j.jcot.2018.11.016 PMC6985028

[22] Yeung E, Jackson M, Sexton S, Walter W, Zicat B, William W (2011) The effect of obesity on the outcome of hip and knee arthroplasty. Int Orthop 35, 929–934. 20512328 10.1007/s00264-010-1051-3 PMC3103952

[23] Collins RA, Walmsley PJ, Amin AK, Brenkel IJ, Clayton RAE (2012) Does obesity influence clinical outcome at nine years following total knee replacement? J Bone Joint Surg 94-B(10), 1351–1355. 10.1302/0301-620X.94B10.28894 23015559

[24] Dowsey MM, Liew D, Stoney JD, Choong PF (2010) The impact of pre-operative obesity on weight change and outcome in total knee replacement: a prospective study of 529 consecutive patients. J Bone Joint Surg Br 92 (4), 513–520. 20357327 10.1302/0301-620X.92B4.23174

[25] Miric A, Lim M, Kahn B, et al. (2002) Perioperative morbidity following total knee arthroplasty among obese patients. J Knee Surg 15, 77–83. 12013077

